# PPAR-delta promotes survival of breast cancer cells in harsh metabolic conditions

**DOI:** 10.1038/oncsis.2016.41

**Published:** 2016-06-06

**Authors:** X Wang, G Wang, Y Shi, L Sun, R Gorczynski, Y-J Li, Z Xu, D E Spaner

**Affiliations:** 1 Biology Platform, Sunnybrook Research Institute, Toronto, Ontario, Canada; 2 Department of Breast Surgery, China-Japan Union Hospital of Jilin University, Changchun, China; 3 Department of Immunology, University of Toronto, Toronto, Ontario, Canada; 4 Transplant Research Division, Toronto General Hospital, Toronto, Ontario, Canada; 5 Department of Anatomy, Norman Bethune College of Medicine, Jilin University, Changchun, China; 6 Department of Medical Biophysics, University of Toronto, Toronto, Ontario, Canada; 7 Sunnybrook Odette Cancer Center, Toronto, Ontario, Canada; 8 Department of Medicine, University of Toronto, Toronto, Ontario, Canada

## Abstract

Expression of the nuclear receptor peroxisome proliferator activated receptor delta (PPARδ) in breast cancer cells is negatively associated with patient survival, but the underlying mechanisms are not clear. High PPARδ protein levels in rat breast adenocarcinomas were found to be associated with increased growth in soft agar and mice. Transgenic expression of PPARδ increased the ability of human breast cancer cell lines to migrate *in vitro* and form lung metastases in mice. PPARδ also conferred the ability to grow in exhausted tissue culture media and survive in low-glucose and other endoplasmic reticulum stress conditions such as hypoxia. Upregulation of PPARδ by glucocorticoids or synthetic agonists also protected human breast cancer cells from low glucose. Survival in low glucose was related to increased antioxidant defenses mediated in part by catalase and also to late AKT phosphorylation, which is associated with the prolonged glucose-deprivation response. Synthetic antagonists reversed the survival benefits conferred by PPARδ *in vitro*. These findings suggest that PPARδ conditions breast cancer cells to survive in harsh microenvironmental conditions by reducing oxidative stress and enhancing survival signaling responses. Drugs that target PPARδ may have a role in the treatment of breast cancer.

## Introduction

A hallmark of lethal breast cancers is their ability to live in metabolic conditions that would otherwise kill normal cells.^1^ This property is associated with resistance to chemotherapy and immunotherapy and ultimately limits patient survival. A better understanding of the mechanisms that allow breast cancer cells to survive in harsh conditions might identify new targets to improve therapeutic outcomes.

The nuclear receptor peroxisome proliferator activated receptor delta (PPARδ) may be a central regulator of the ability of cells to thrive in harsh conditions. It is the least characterized of the nuclear receptor family that includes PPARγ and PPARα, which control fat storage in adipocytes and fatty acid oxidation in liver and muscle, respectively.^2^ PPARδ is expressed ubiquitously and, in the absence of ligands, binds corepressors like NCOR1 and recruits histone deacetylases to repress gene expression. PPARδ is activated by high concentrations of free fatty acids, bioactive lipids and synthetic agonists such as GW501516 and GW0742.^2^ Following ligand binding, it undergoes a conformational change and mediates transcription of genes such as *PPARD* itself, *ANGPTL*4 and antioxidant genes such as *CAT* (catalase) that serve as ‘signatures' for PPARδ activity.^3^

PPARδ increases the endurance capacity of muscle cells^4^ and prevents exhaustion of hematopoietic stem cells by lowering oxidative stress and preventing symmetric cell divisions.^5, 6^ For success in these situations, cells are required to function effectively over relatively long periods of time in the presence of increasingly unfavorable metabolic conditions. If PPARδ had similar activity in cancer cells as in muscle and stem cells, it could allow them to grow in metabolically stressful conditions.^1, 7^ We have shown that PPARδ mRNA and protein expression are upregulated when glycolysis is inhibited in leukemia cells.^8^ The experiments in this manuscript were designed to investigate the effect of PPARδ in harsh conditions such as found in breast cancer microenvironments.^9^

## Results

### PPARD upregulation in breast cancer cells is associated with more aggressive clinical behavior

**Figure 1:**
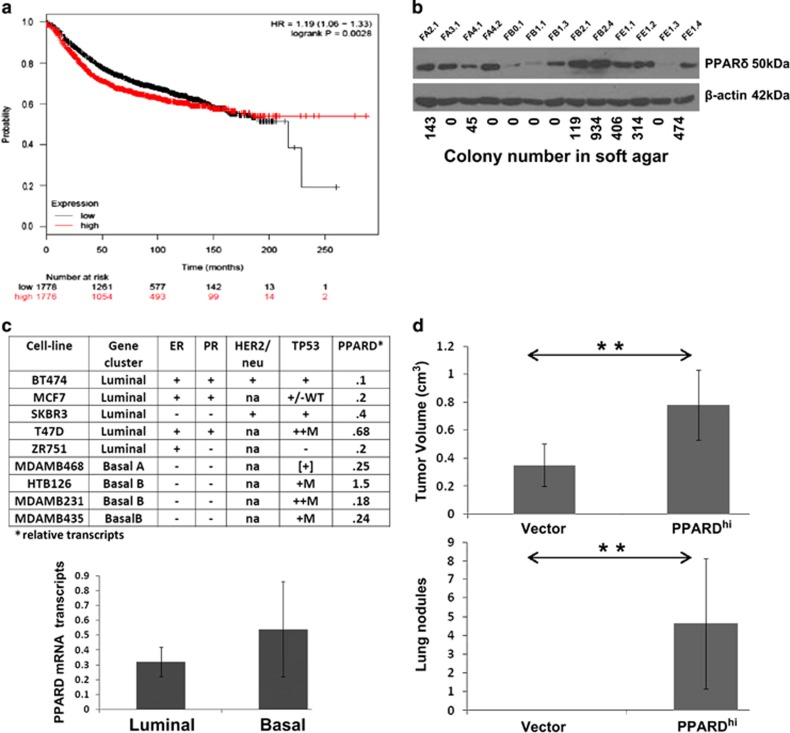
Association of PPARδ expression with aggressive behavior of breast cancer cells. (**a**) Overall survival of 2500 breast cancer patients as a function of *PPARD* gene expression in their biopsies. (**b**) PPARδ expression by immunoblotting in clones of rat mammary adenocarcinomas with β-actin used as a loading control. Numbers of colonies from plating 5 × 10^3^ cells in soft agar are shown for each clone.^12^ (**c**) *PPARD* expression was measured by RT–PCR in the nine human breast cancer cell lines described in the table. The average and standard error of *PPARD* expression for the basilar and luminal cell lines is shown in the bottom graph. (**d**) Two groups of NSG mice (*n*=5) were injected in the mammary fat pad with MCF-7 cells transfected with either a *PPARD* expression vector (clone 7 with high *PPARD* expression) or the vector alone. Mice were killed after 21 days and local tumor volumes measured with calipers. Numbers of tumor colonies in the lungs were determined by visual inspection. **P<0.05.

The magnitude of *PPARD* expression in 295 different breast cancer samples has been associated directly with overall survival.^10^ We confirmed this by analyzing a public database of over 2500 clinically annotated breast cancer samples^11^ (Figure 1a).

Previously, we characterized a number of clones of adenocarcinomas derived from rats that had been injected with v-Ha-Ras transgene-expressing retroviruses into the mammary ducts. The ability of these clones to grow in soft agar was shown to be predictive of aggressive behavior *in vivo*.^12^ Expression of PPARδ by these clones was measured by immunoblotting (Figure 1b). PPARδ levels were low or undetectable in four out of six of the non-aggressive clones that did not grow in soft agar. In contrast, all seven aggressive clones that grew well in soft agar expressed high PPARδ (Figure 1b).

A panel of luminal and basilar breast human cancer cell lines^13^ was then screened for *PPARD* mRNA expression (Figure 1c). There was a trend toward higher expression of *PPARD* in lines derived from basilar breast cancers, which are considered to have more aggressive clinical behavior.^14^

MCF-7 cells were then used to study the effects of increasing *PPARD* expression as they had relatively low baseline mRNA expression (Figure 1c). The cells were transfected with retroviruses expressing human *PPARD* and clones of PPARD^hi^-MCF-7 cells were generated as described in the materials and methods. PPARD^hi^ and control MCF-7 cells transfected with expression vectors alone were then injected into the mammary fat pads of NSG female mice. After 21 days, PPARD^hi^-MCF-7 cells exhibited higher local growth and metastasized to the lungs to a greater extent, consistent with more aggressive behavior (Figure 1d).

### PPARδ increases survival of MCF-7 cells in low extracellular glucose

**Figure 2:**
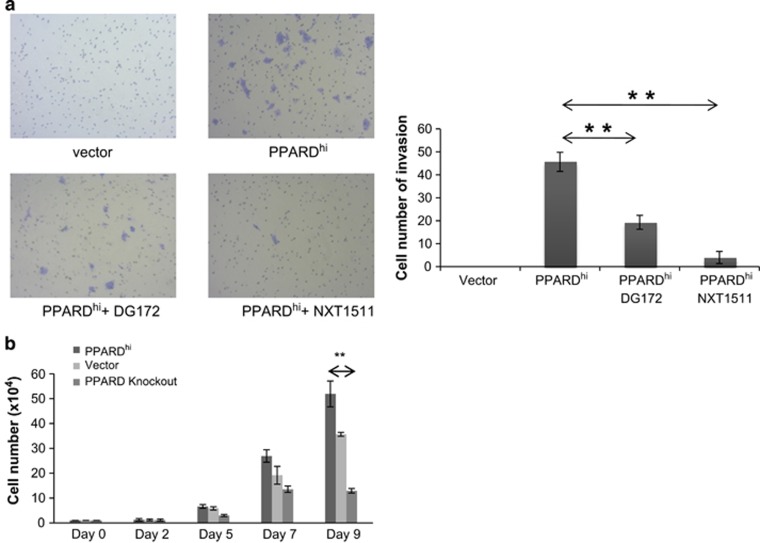
Migration and growth of PPARD^hi^, knockout and control MCF-7 cells in conventional glucose conditions. (**a**) Transwell invasion assays were performed as described in the materials and methods in the presence or absence of the PPARD antagonists DG172 or NXT1511 (3 μM). Cells that migrated to the bottom of the insert were counted after 96 h. Pictures of the stained and fixed inserts are shown in the upper panels ( × 10 magnification) and the average and standard error of the numbers of large, blue migrated cells are shown in the lower graph. (**b**) Cells were plated at an initial concentration of 10^4^ cells/ml in 24-well plates in DMEM+5% FBS and counted manually on the indicated days. The average and standard error of the results of three different counts per well are shown. Experiments were repeated three times with similar results. **P<0.05.

Consistent with the increased propensity to metastasize *in vivo*, PPARD^hi^-MCF-7 cells also exhibited greater migration *in vitro* in response to chemotactic factors in fetal bovine serum (FBS) (Figure 2a). PPARD^hi^-MCF-7 cells did not grow much differently than control cells for the first few days of culture in conventional conditions (Dulbecco's modified Eagle's media (DMEM)+5% FBS). However, if the cultures were continued without feeding, PPARD^hi^ cells grew better and there were significantly more PPARD^hi^ cells by day 9 than control MCF-7 cells (Figure 2b).

*PPARD* was not completely absent from the control cells, although it was expressed to a much lower extent than in PPARD^hi^ cells. PPARδ knockout cells were generated by CRISPR/Cas9 technology, as described in the materials and methods. These cells grew more slowly and their numbers at day 9 were much lower than both PPARD^hi^ cells and control MCF-7 cells (Figure 2b).

**Figure 3:**
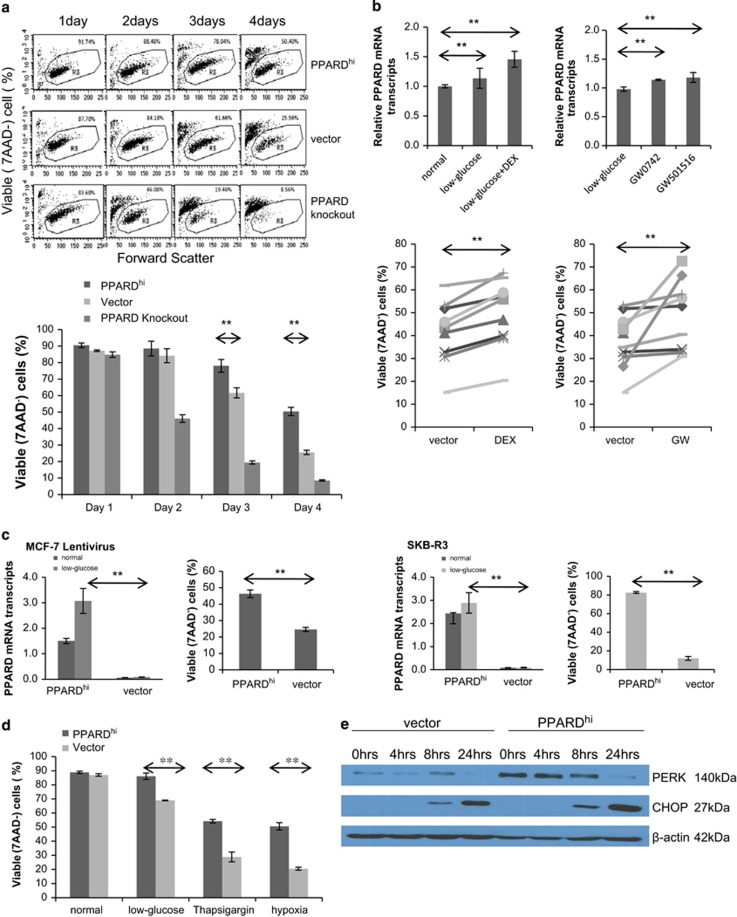
Survival of breast cancer cells in low glucose and other harsh conditions as a function of PPARD expression. (**a**) Control, PPARD^hi^ and PPARD knockout MCF-7 cells were cultured in glucose-free RPMI+5% non-dialyzed fetal calf serum. Percentages of viable cells that excluded 7-aminoactinomycin D (7AAD) were then determined at the indicated times by flow cytometry. The numbers in the scatter plots are the percentages of viable 7AAD^−^ cells. Averages and standard errors from three different experiments are shown in the lower graph. (**b**) Control MCF-7 cells were cultured in low-glucose conditions with or without dexamethasone (DEX) (30 μM) (left panels) or GW0742 (1 μM) or GW50516 (100 nM) (right panels) to increase *PPARD* expression. *PPARD* (top panels) and percentages of 7AAD^−^ cells (bottom panels) were measured by RT–PCR or flow cytometry after 72 h. Each line represents the results from a different experiment and the average of all experiments was used for statistical calculations. Results with the two synthetic agonists were pooled, as indicated by GW on the *x* axis of the right bottom graph. (**c**) MCF-7 cells infected with lentiviruses (left panel) or SKB-R3 cells infected with retroviruses (right panel) expressing *PPARD* or the vectors alone were cultured in low-glucose conditions. Viable cells were determined by flow cytometry after 3 days. The average and standard errors of two separate experiments are shown. *PPARD* expression by RT–PCR under normal and low-glucose conditions after 2 days is shown in the other graphs. (**d**) Viable cells were determined after 2 days of culture in conventional conditions with or without thapsigargin or hypoxia or in low-glucose conditions. Averages and standard errors of three separate experiments are shown. (**e**) Cells were cultured in low-glucose conditions and levels of PERK and CHOP were determined at the indicated times by immunoblotting using β-actin as a loading control. **P<0.05.

After 9 days without feeding, the culture media is expected to represent harsh metabolic conditions as the cells use up nutrients such as glucose.^15^ On the basis of their behavior in continuous culture (Figure 2b), PPARD^hi^ cells were tested for their ability to survive directly in low-glucose conditions. PPARD^hi^, control and knockout cells in DMEM+5% FBS (4.5 gm/l=25 mM glucose) were washed and cultured in glucose-free RPMI+5% non-dialyzed FBS (0.25 mM glucose) and cell viability was determined at various times (Figure 3a). After 2–3 days, survival of PPARD^hi^ cells was much better than controls, whereas PPARD knockout cells did quite poorly in these harsh conditions (Figure 3a).

To determine whether induction of *PPARD* expression without genetic manipulation also conferred the ability to survive in low-glucose conditions, MCF-7 cells were treated with the glucocorticoid receptor agonist dexamethasone (30 μM) or the PPARδ agonists GW0742 and GW501516. This concentration of dexamethasone promotes PPARD expression in leukemia cells^8^ and PPARδ is known to auto-regulate itself.^16^ *PPARD* mRNA levels increased modestly in low-glucose conditions and were also increased by dexamethasone and the PPARδ agonists (Figure 3b, top panels). Consistent with the increase in *PPARD*, the cells also survived to a greater extent upon culture in low-glucose conditions (Figure 3b, bottom panels).

To determine whether this ability to survive in low-glucose was directly related to *PPARD* overexpression and not acquired because of selection processes from prolonged tissue culture, MCF-7 cells were independently transfected with lentiviruses that expressed *PPARD* (Figure 3c, left panels) along with a turbo-red fluorescent gene. Owing to infection efficiencies of 50% or more and the ability to sort *PPARD*-expressing cells by flow cytometry, this method allowed PPARD^hi^-MCF cells to be studied within a few days of infection. PPARD^hi^-MCF-7 cells made with lentiviruses also survived much better and further increased their expression of *PPARD* in low glucose, suggesting these properties were conferred directly by PPARδ (Figure 3c, left panels).

To determine that this ability to survive in low glucose was not specific to MCF-7 cells, SKB-R3 breast cancer cells were also transfected with *PPARD*. Clones of PPARD^hi^-SKB-R3 cells also acquired the ability to survive in low-glucose compared with cells transfected with the vector alone. *PPARD* levels also increased further upon culture in low-glucose conditions (Figure 3c, right panels).

### PPARδ protects MCF-7 cells from endoplasmic reticulum (ER) stress

Glucose deprivation causes an ER stress response^17^ and PPARδ has been shown to protect cells from developing ER stress.^18^ Consistent with this, PPARD^hi^-MCF-7 cells also survived better than control cells in other conditions that cause ER stress including treatment with thapsigargin and hypoxia (Figure 3d).

The unfolded protein response is activated by ER stress and mediated by PERK, ATF6 and IRE1.^19^ PERK causes a transcriptional block that facilitates transcription of activating transcription factor 4 (ATF4), which mediates transcription of the gene encoding the proapoptotic protein CHOP.^19^ Baseline expression of PERK appeared to be higher in PPARD^hi^-MCF-7 cells but disappeared after 24 h of glucose deprivation (Figure 3e). Expression of CHOP began to increase after 8 h of glucose deprivation in both cell lines and was even higher in PPARD^hi^-MCF-7 cells (Figure 3e). These findings suggest that PPARδ did not prevent the development of ER stress but protected breast cancer cells from the consequences of ER stress.

### PPARδ protects MCF-7 cells from the oxidative stress of glucose deprivation

**Figure 4:**
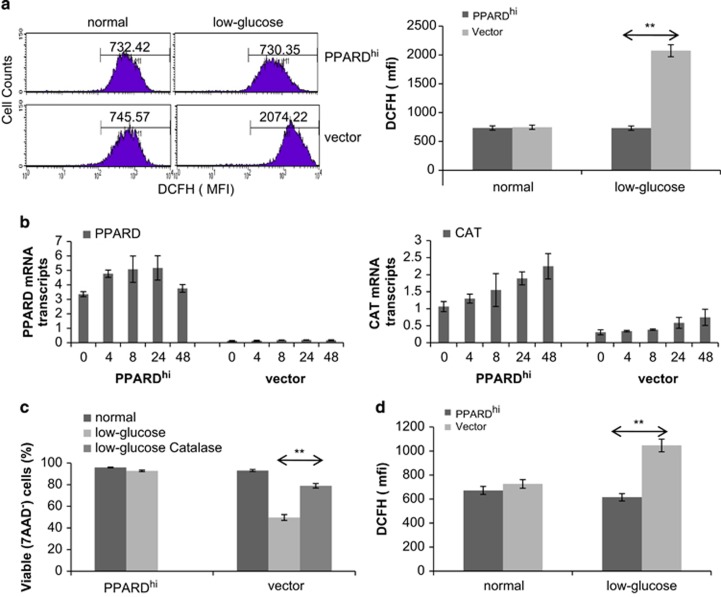
Effect of PPARδ on glucose deprivation-induced oxidative stress and catalase expression. (**a**) PPARD^hi^ and vector control MCF-7 cells were cultured in normal or low-glucose conditions for 24 h and then stained with DCFH as a measure of reactive oxygen species (ROS) levels. Examples of the flow cytometric analyses are shown on the left. The numbers in the histograms represent the mean fluorescence intensity (MFI) of DCFH staining. Averages and standard errors of three separate measurements are shown on the right. (**b**) MCF-7 cells were cultured in low-glucose condition for 0, 4, 8, 24 and 48 h. *PPARD* and *CAT* levels were measured at these times by RT–PCR. (**c**) The cells were cultured in low-glucose with or without catalase (20 μg/ml) for 3 days and the percentages of viable 7-aminoactinomycin D (7AAD)^−^ cells were then determined by flow cytometry. (**d**) MCF-7 cells infected with PPARD-expressing or control lentiviruses were cultured in normal or low-glucose conditions for 24 h and then stained with DCFH. Averages and standard errors of two to three separate measurements are shown. **P<0.05.

Glucose deprivation is known to cause oxidative stress in cancer cells^20^ and PPARδ has been shown to increase the antioxidant defenses of a number of cell types, including neurons and cardiomyocytes.^21, 22^ Oxidative stress increased considerably in control MCF-7 cells after 24 h in low-glucose conditions as measured by staining with dichloro-dihydro-fluorescein diacetate (DCFH), which indicates levels of reactive oxygen species.^23^ In contrast, DCFH staining did not change significantly in PPARD^hi^-MCF-7 cells (Figure 4a), suggesting they were protected from oxidative stress. Short-term expression of *PPARD* in MCF-7 cells by lentiviruses also conferred protection from oxidative stress (Figure 4d), suggesting it was caused directly by PPARδ and not simply an epiphenomenon associated with increased *PPARD* expression.

Catalase is one of the antioxidant genes that is regulated by PPARδ.^24^ To determine whether it might help PPARD^hi^-MCF-7 cells resist oxidative stress, catalase (*CAT*) and *PPARD* expression were measured at serial times over 48 h of culture in low glucose (Figure 4b). *CAT* mRNA levels increased more than twofold in both vector control and PPARD^hi^ cells, beginning around 8 h after glucose deprivation (Figure 4b). Because *CAT* expression was initially much higher in PPARD^hi^ cells, *CAT* mRNA levels remained fourfold to fivefold higher in PPARD^hi^ cells for at least 48 h (Figure 4b). Similar results were seen for *PPARD* itself. Addition of exogenous catalase to correct the defect in *CAT* expression partially rescued control MCF-7 cells from glucose starvation (Figure 4c).

### PPARδ increases the AKT-mediated survival response to severe glucose deprivation

**Figure 5:**
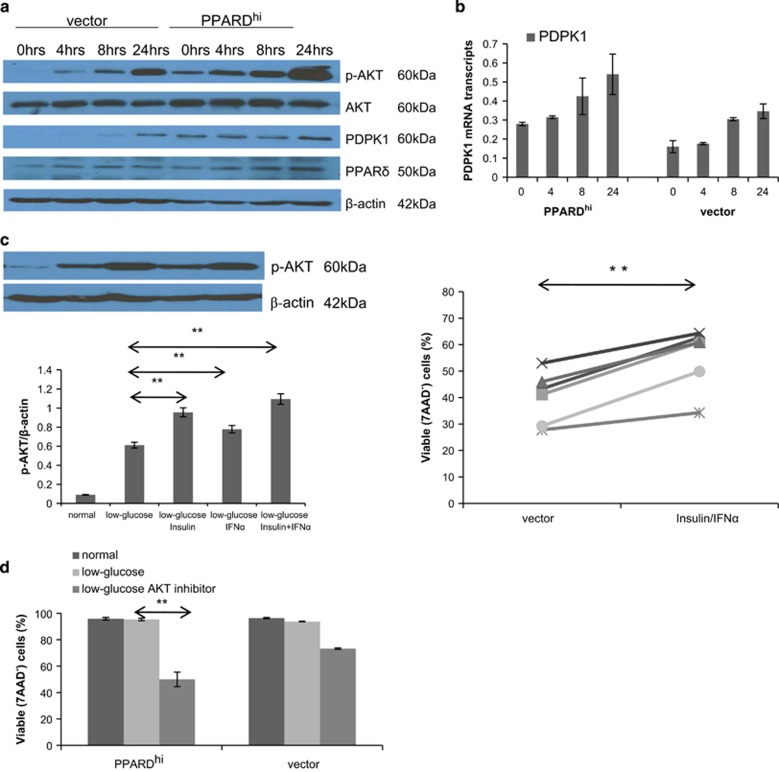
Effect of PPARδ on the phosphorylation of AKT induced by prolonged glucose deprivation. (**a**, **b**) Control and PPARD^hi^-MCF-7 cells were cultured in low-glucose conditions. Protein extracts were made at 0, 4, 8 and 24 h and phospho-AKT, AKT, PDPK1 and PPARδ levels were determined by immunoblotting with β-actin as a loading control (**a**). PDPK1 mRNA levels were measured at these times by RT–PCR (**b**). (**c**) Control MCF-7 cells were cultured in low-glucose conditions with insulin (0.1 IU) and/or IFNα (1000 IU). After 24 h, p-AKT levels were quantified by immunoblotting (left top panel) and densitometry relative to β-actin (left bottom graph). Viable 7-aminoactinomycin D (7AAD)^−^ cells were measured after 3 days (right graph). Each line shows the result from a separate experiment. (**d**) PPARD^hi^ and control MCF-7 cells were cultured with or without AKT inhibitor IV (0.2 μM) in low-glucose conditions. Percentages of viable 7AAD^-^ cells were measured after 2 days. Averages and standard errors of two to three separate measurements are shown. **P<0.05

The serine/threonine kinase AKT is another important mediator of cancer cell survival in low-glucose conditions.^25, 26^ Short-term glucose deprivation (less than 6 h) causes a modest increase in AKT phosphorylation owing to a release of feedback inhibition from p70S6K. Prolonged glucose deprivation (>16 h) induces a marked increase in AKT phosphorylation owing to the formation of a complex that consists of AKT together with the heat shock protein GRP78 and the AKT-activating kinase PDPK1.^25^ Accordingly, phospho-AKT levels were measured over time in MCF-7 cells in low-glucose conditions (Figure 5a). Consistent with the survival benefit conferred by *PPARD* overexpression in these conditions (Figure 2), phosphorylation of AKT was much higher in PPARD^hi^-MCF-7 cells at 24 h compared with vector control cells (Figure 5a).

PPARδ has been shown to regulate the AKT signaling pathway and *PDPK1* is a transcriptional target of PPARδ.^27^ PDPK1 mRNA (Figure 5b) and protein (Figure 5a) levels increased over time in both control and PPARD^hi^-MCF-7 cells. However, PDPK1 levels were higher initially and also at 24 h in PPARD^hi^-MCF-7 cells, potentially accounting for the greater phosphorylation of AKT (Figure 5a).

PPARD^lo^ vector control cells were then treated with insulin, IFN-alpha or both at 0 and 24 h after culture in low glucose (Figure 5c) to activate AKT at later times and implicate the changes in AKT phosphorylation with survival. The combination of IFN-alpha and Insulin increased p-AKT levels (Figure 5c, left panel) in these cells and their survival after 3 days (Figure 5c, right panel). Conversely, inhibition of AKT with a small molecule inhibitor decreased the survival of both vector control and PPARD^hi^ MCF-7 cells in low glucose. PPARD^hi^ cells were especially sensitive to AKT inhibition in these conditions (Figure 5d).

### Small molecule inhibitors reverse the survival effects of PPARδ

Despite the apparent importance of PPARδ in cancer biology, there are presently no PPARδ antagonists available for clinical use. However, a number of recent tool compounds^28, 29, 30, 31, 32^ can inhibit activation of *PPARD* reporter constructs by ligands with EC50s in the 10–100 nM range and little cross-reactivity to other nuclear receptors. DG172 exhibits high binding affinity and potent inverse agonistic properties^28^ (that is, binds PPARδ as an agonist but decreases basal expression of target genes by increasing recruitment of corepressors). PT-S58 is a cell-permeable-specific competitive antagonist, targeting the ligand-binding site of PPARδ but not allowing coregulator interactions.^30^ NXT1511 is another chemical compound that inhibits PPARδ at low micromolar concentrations.^33^

**Figure 6:**
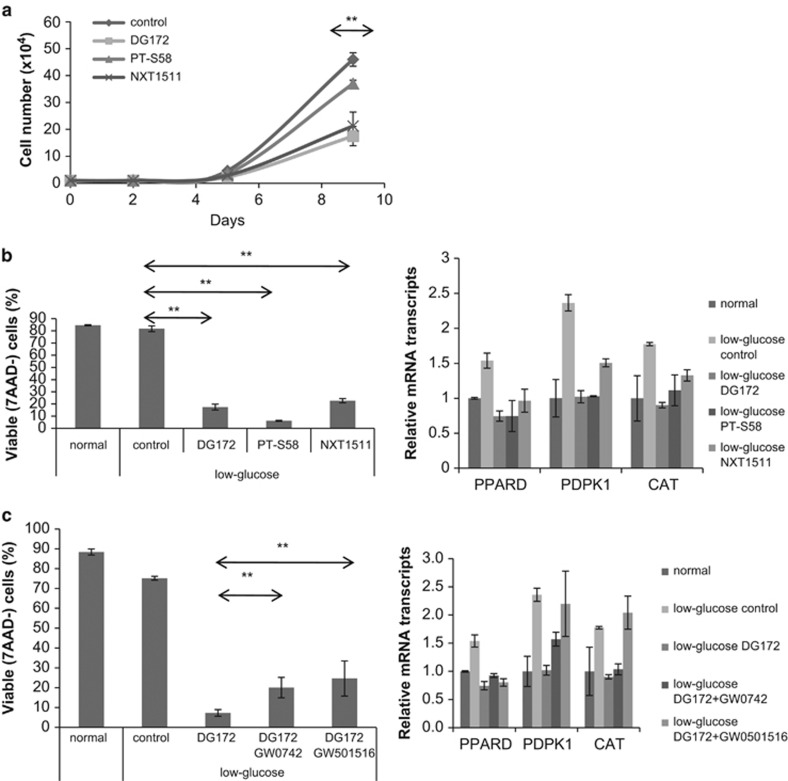
Effect of PPARδ antagonists on cell growth and survival. (**a**) PPARD^hi^-MCF-7 cells were cultured in DMEM+ 5% FBS medium with or without the PPARδ antagonists DG172 (3 μM), PT-S58 (30 μM) or NXT1511 (3 μM). Viable cells were counted manually at the indicated times. (**b**, **c**) PPARD^hi^-MCF-7 cells maintained in normal glucose conditions were transferred to low-glucose conditions in the presence or absence of the PPARD antagonists DG172, PT-S53 or NXT1511 (**b**) or with the PPARδ agonists GW0742 (1 μM) or GW501516 (100 nM) along with DG172 (3 μM) (**c**). Expression of *PPARD* and its signature genes *PDPK1* and *CAT* were measured by RT–PCR after 24 h (right panels). Percentages of viable 7-aminoactinomycin D (7AAD)^−^ cells were determined by flow cytometry after 3 days (left panels). Averages and standard errors of two to three separate measurements are shown. **P<0.05

All of these inhibitors decreased the ability of PPARD^hi^-MCF-7 cells to grow in exhausted tissue culture media (Figure 6a). The ability of these cells to survive following glucose deprivation was also reversed (Figure 6b, left panel) along with the upregulation of the PPARδ-regulated genes *PPARD*, *PDPK1* and *CAT* that occurs in low glucose (Figure 6b, right panel). Invasion of PPARD^hi^-MCF-7 cells was also inhibited by DG172 and NXT1511 (Figure 2a).

The fact that three different chemical inhibitors of PPARδ gave the same results provided some assurance that the results could be explained by inhibition of PPARδ. To provide additional evidence that the effects of the inhibitors were not simply due to off-target activity, the PPARδ synthetic agonists GW0742 and GW501516 were used.^2^ As DG172 is thought to bind to the ligand-binding site of PPARδ, it should be displaced by these synthetic agonists. Both agonists partially increased the survival of PPARD^hi^ cells in the presence of DG172 in low-glucose conditions (Figure 6d, left panel) along with *PDPK1* and *CAT* mRNA expression (Figure 6d, right panel). GW501516 appeared to be more potent in this regard than GW0742.

## Discussion

The results in this manuscript indicate that *PPARD* is expressed by breast cancer cells with more aggressive clinical behavior (Figure 1). Higher PPARδ levels confer increased migratory (Figure 2a) and metastatic (Figure 1d) properties along with the ability to survive in harsh metabolic conditions such as exhausted tissue culture media (Figure 2b) or low glucose (Figure 3). PPARδ mediates these effects by mechanisms that include increased expression of antioxidant proteins such as catalase (Figure 4) and enhanced AKT-mediated survival signaling after prolonged nutrient deprivation (Figure 5). PPARδ-antagonist tool compounds can reverse these effects *in vitro* (Figure 6).

The role of PPARδ in cancer biology appears to be context-dependent. PPARδ can prevent tumors, perhaps through anti-inflammatory effects, but it promotes angiogenesis and progression of cancers once they are established.^34, 35, 36, 37^ Clinical evidence supports an association of PPARδ with aggressive cancers. For example, PPARδ expression is inversely correlated with survival in gastrointestinal cancers.^38^ Consistent with our findings (Figure 1), PPARδ has been implicated as an important transcriptional node in breast cancer, and shorter survival of breast cancer patients is associated with increased expression of PPARδ by their tumor cells (Figure 2).^10^ Synthetic PPARδ ligands also promote breast cancer progression and metastasis in transgenic mice.^37^

Our results suggest PPARδ allows breast cancer cells to ‘endure' harsh metabolic conditions (Figures 2,3 and 4), analogous to its ability to promote endurance in muscle cells and prevent exhaustion of stem cells.^4, 5, 6^ Taken together, the observations suggest that PPARδ drives aggressive clinical behavior because it allows cancer cells to grow in metabolically stressful conditions, which would include the presence of chemotherapies that cause ER stress (Figure 3d).^1, 7, 8^

It is not entirely clear why PPARδ should be expressed by aggressive cancers. *PPARD* is located at chromosome 6p21.2, which is a site of gain in estrogen receptor-negative and high-risk breast cancers.^39^ However, *PPARD* appears to be expressed mainly in response to factors in the microenvironment such as glucocorticoids (Figure 3b), cytokines^40^ and signals that activate calcineurin.^41^ We found it was also increased by low extracellular glucose levels (Figures 3b and 4b) that cause ER stress (Figure 3d). Interestingly, the transcription factor ATF4 is expressed in ER stress conditions^19^ and may co-regulate the expression of PPARδ-regulated genes, which include *PPARD* itself.^3^ However, transcription of PPARδ-regulated genes did not seem to absolutely require concomitant ER stress as baseline levels were higher in PPARD^hi^ cells growing in high-glucose ‘stress-free' conditions (Figures 4 and 5). *CAT* and *PDPK1* gene expression did increase following glucose deprivation to protect PPARD^hi^ MCF-7 cells from glucose stress (Figures 4 and 5), but also increased in control cells although presumably not to sufficient levels to mediate protection from the harsh conditions. In contrast, higher baseline levels of these genes in PPARD^hi^ cells meant that even higher levels were achieved following glucose deprivation. Thus, high levels of PPARδ appear to ‘condition' the cells to survive in harsh conditions.

Synthetic agonists of PPARδ also increased *PPARD* levels in MCF-7 cells (Figures 3b). Natural ligands of PPARδ include bioactive lipids such as prostacyclin,^42^ 15-HETE^43^ and 5-Oxo-ETE,^44^ derived from arachidonic acid by cyclooxygenase and lipoxygenase enzymes. Other PPARδ ligands include high concentrations of free fatty acids released from lipoproteins by lipoprotein lipase^45^ or intracellular lipid droplets by ATGL.^46^ It is unclear whether any of these ligands are activating PPARδ in PPARD^hi^-MCF-7 cells or if sources of activating ligands change in different microenvironmental conditions and mediate different outcomes.

There are presently no clinically relevant PPARδ antagonists, but existing drugs may block some of the effects of PPARδ. For example, AKT inhibitors have been proposed to overcome the late survival signaling responses that allow some cancer cells to survive prolonged glucose deprivation.^25^ If PPARδ regulates this response (Figure 5), then AKT inhibitors may act downstream of PPARδ to kill tumor cells. However, protection by PPARδ appears to involve multiple mechanisms, including prevention of oxidative stress (Figure 4), which would not necessarily be blocked by ATK inhibitors and could help explain the weak effects of these agents in clinical trials.^47^ The results with tool compounds (Figure 6) suggest they may be used to engineer clinically relevant anti-PPARδ drugs. An alternative might be to use lipase inhibitors and combinations of clinically relevant lipoxygenase and cyclooxygenase inhibitors to block ligand generation and prevent the activation of PPARδ.^48, 49^ On the basis of the apparent importance of PPARδ in mediating the behavior of aggressive breast cancer cells, it would appear that strategies to target this nuclear receptor may ultimately improve the outcomes of breast cancer patients.

## Materials and methods

### Cell line and cell culture

The human breast cancer cell lines MCF-7, SKB-R3 and other lines described in Figure 1c were obtained from American Type Culture Collection (ATCC). Rat breast cancer cells shown in Figure 1b have been previously described.^12, 50^

Cells were cultured in high-glucose DMEM (Multicell) or glucose-free RPMI 1640 Media (Multicell, Toronto, ON, Canada) supplemented with 5% FBS and 1% penicillin-streptomycin (Multicell) at 37 °C with 5% carbon dioxide.

### Antibodies and reagents

PT-S58 (PPARδ antagonist), catalase, thapsigargin and β-actin antibodies were from Sigma-Aldrich (St Louis, MO, USA). DCFH was from Life-Tech (Carlsbad, CA, USA) while 7-aminoactinomycin D was from Biolegend (San Diego, CA, USA). GW0742 and GW501516 (PPARδ agonists) were from Cayman Chemical (Ann Arbor, MI, USA). Dexamethasone (Omega, Montreal, QC, Canada), insulin (Eli Lilly, Toronto, ON, Canada) and interferon-α2b (Schering Canada Inc., Pointe-Claire, QC, Canada) were purchased from the hospital pharmacy. AKT inhibitor IV was from Calbiochem (San Diego, CA, USA). DG172 (PPARδ antagonist) has been previously described.^28^ NXT1511 (PPARδ antagonist) was provided by Peppi Prasit (Inception, San Diego, CA, USA).

Antibodies to PERK, PDPK1, p-AKT(T308), AKT, p-SAPK/JNK (T183/Y185), CHOP, anti-Rabbit IgG and anti-Mouse IgG were from Cell Signaling Technology (Danvers, MA, USA). The PPARδ antibody (101720) was from Cayman.

### Retroviral and lentiviral infections

Human PPARD full cDNA was obtained from Addgene (Cambridge, MA, USA) and sub-cloned into the *Xho*I and *Eco*RI sites of retroviral MSCV2.2 plasmids or into the *Xho*I and *Not*I sites of lentiviral pLemiR plasmids. Sequences of the constructs were confirmed before transfection. Replication-defective viruses were prepared by transfecting the MSCV-PPARD viral plasmid into the helper-free packaging cell line GP+A (B8), as described previously.^50^ Supernatants from the virus-producing cells were used to infect MCF-7 and SKB-R3 cells, plated at a density of 2 × 10^6^ cells/ml. Stably transfected clones were obtained by limiting dilution and selection in G418 (Multicell). Transfection was conducted with Lipofectamine 3000 according to the manufacturer's protocol (Invitrogen, Carlsbad, CA, USA). Cells infected with retroviruses containing the empty vectors but otherwise handled in the same way were used as controls.

To make lentiviruses, 8 × 10^5^ HEK293T cells were seeded into 6-well plates and transfected 24 h later with plemiR-PPARδ plasmids (1 μg) and package plasmids (0.8 μg 8.2VPR vector and 0.2 μg VSVG vector) using Lipofectamine 3000 according to the manufacturer's instructions. After 24 h, the media was replaced with 2 ml fresh media. After 48 h, the supernatants containing lentivirus particles were collected and used to infect MCF-7 and SKB-R3 cells. Infected cells expressed turbo-red fluorescent proteins and were sorted on a flow cytometer. Control cells were also made with the empty plasmids.

### Generation of PPARδ knockout cells

To generate PPARδ loss-of-function phenotypes, *PPARD* was targeted by commercial pLV-U6g-EPCG CRISPR single plasmids (HS0000171233, Sigma), containing the PPARD gRNA and Cas9 element. MCF-7 cells (7 × 10^5^) were seeded into 6-well plates and transfected with 2 μg lentiviral pLV-U6g-EPCG-PPARδ CRISPR plasmid using Lipofectamine 3000. After 24 h, stably infected cells were selected by growth in puromycin at 1 μg/ml for a week. Successful knockout of PPARD was confirmed by immunoblotting.

### Hypoxia treatment

MCF-7 cells were cultured at 1 × 10^6^ cells/well in 6-well plates or 2 × 10^4^ cells/well in 24-well plates in high-glucose DMEM with 5% FBS in an INVIVO_2_ 200 hypoxia workstation (Ruskinn, Bridgend, Mid Glamorgan, UK) that was flushed with a mixture of 1 O_2_, 5 CO_2_ and 94.5% N_2_. Anaerobic conditions were confirmed by using a Hypoxia Gas Mixer Q (Ruskinn) to read the O_2_ content in the workstation.

### Cell proliferation assays

Breast cancer cells were seeded at a density of 10^4^ cells/well in 24-well culture plates and counted in a hemocytometer at days 2, 5, 7 and 9.

### Isolation of RNA and synthesis of cDNA

MCF-7 and SKB-R3 cells were harvested and washed. Total RNA was extracted using the RNeasy kit (Qiagen, Mississaga, ON, Canada) according to the manufacturer's instructions. RNA concentrations were determined in a spectrophotometer at 260 nm.

Subsequent cDNA synthesis was performed using the Superscript III First Strand Synthesis System for RT–PCR (Invitrogen) in a 20-μl reaction containing 2 μg total RNA, 20 mM Tris-HCl (pH 8.4), 2.5 mM MgCl2, 5 mM dithiothreitol, 2.5 μm OligodT20, 0.5 mM each of dATP, dGTP, dCTP, dTTP and 200U Superscript III Reverse Transcriptase. The priming oligonucleotide was annealed to total RNA by incubating at 65 °C for 5 min and cooling to 4 °C. Reverse transcription was performed at 50 °C for 50 min and cDNA was stored at −20 °C until used for real-time PCR analysis.

### Real-time PCR

The following primers were used to amplify human *PPARD, PDPK1, CAT* and *HPRT* transcripts: PPARD forward: 5′-CTCTATCGTCAACAAGGACG-3′ reverse: 5′-GTCTTCTTGATCCGCTGCAT-3′. PDPK1 forward: 5′-TAACAAGAGAGCGGGATGTC-3′; reverse: 5′-ATCGGGTACAGGTCTCATCG-3′. Catalase forward: 5′-CCTTTCTGTTGAAGATGCGGCG-3′ reverse: 5′-GGCGGGTGAGTGTCAGGATAG-3′. HPRT forward: 5′-GAGGATTTGGAAAGGGTGTT-3′ reverse: 5′-ACAATAGCTCTTCAGTCTGA-3′. PCR was performed on a DNA engine Option System (MJ Research Inc, Waltham, MA, USA) using SYBR Green (Life Technologies, Warrington, UK) as a double-stranded DNA-specific binding dye. PCR reactions were cycled 40 times after initial denaturation (95 °C, 15 min) with the following parameters: denaturation at 95°C for 20 s, annealing of primers at 58 °C for 20 s, and extension at 72 °C for 20 s. Fluorescent data were acquired during each extension phase. After each PCR reaction, a melting curve analysis of amplification products was performed by increasing the temperature to 95 °C at 0.2 °C/s. Fast loss of fluorescence is observed uniquely at the denaturing/melting temperature of the amplified DNA fragment. Standard curves were generated with serial 10-fold dilution of cDNAs obtained with the same primers as for real-time PCR.

### Western blots

MCF-7 cells were collected and lysed for 30 min in lysis buffer (0.5% TritonX-100, 25 mM MES, 150 mM NaCl, 1 mM Na_3_VO_4_, 2 mM EDTA, 1 mM PMSF, 1 μg/ml aprotinin) at 4 °C, followed by high-speed centrifugation for 15 min. Protein extracts were collected, quantified by the method of Bradford and prepared for immunoblotting by 1:4 dilution in 5 × sample buffer (8% (wt/vol) SDS, 8% (vol/vol) 2-ME, 250 mM Tris, 40% glycerol, 2% bromophenol blue in dd-H_2_O) and denaturation at 100 °C for 5 min. Sample were then loaded on a discontinuous polyacrylamide gel consisting of 10% resolving and 5% stacking gels. The separated proteins were then transferred to Immobilon-P membranes (EMD Millipore, Billerica, MA, USA) that were pre-activated with 100% methanol. Blots were blocked with 5% milk or bovine serum albumin for 1 h before incubation with primary antibodies followed by anti-rabbit or anti-mouse antibodies. Signals were detected with Supersignal horseradish peroxidase enhanced chemiluminescence reagent (Thermo Fisher Scientific, Waltham, MA, USA), and blots were exposed to premium autoradiography film. Blots were stripped for 60 min at 37 °C in Restore Western blot Stripping buffer (Thermo) washed twice in Tris-buffered saline plus 0.05% Tween-20 at room temperature, blocked and re-probed, as required. Antibodies to β-actin (1:50 000 dilution) were used to control for loading.

### Flow cytometric analysis of live cells and reactive oxygen species

Cells were transferred to conical tubes, pelleted and resuspended in 500 μl phosphate-buffered saline with 3 μl 7-aminoactinomycin D. After 15 min in the dark at room temperature, the cells were analyzed on a FACSCalibur flow cytometer (BD Biosciences, Mississauga, ON, Canada) using CellQuest flow. At least 10 000 events were collected for each experiment.

The dye 2'7'-dichlorofluorescin diacetate (DCFH_2_-DA) (Molecular Probes, Eugene, OR, USA) was used to indicate intracellular reactive oxygen species formation. Intracellular esterases cleave the acetyl groups from the molecule to produce non-fluorescent DCFH_2_, which is trapped inside the cell. In the presence of reactive oxygen species, DCFH_2_ is oxidized to DCF, which emits fluorescence at 530 nm, after excitation at 488 nm. Breast cancer cells were incubated with 10 μM DCFH_2_-DA at 37 °C for 30 min. Samples were then washed in phosphate-buffered saline and DCFH_2_ oxidation was measured as ‘green' (FL1) fluorescence on a log scale for 10 000 events.

### Transwell cell invasion assay

Transwell 24-well chambers (Corning, NY, USA) were used to monitor cell invasion. The upper side of the filter was covered with Matrigel (Corning). DMEM with 5% FBS containing chemoattractants was added to the lower chamber. MCF-7 cells (1 × 10^5^ cells in 100 μl DMEM alone) were plated in the upper chamber and incubated at 37 °C for 96 h. Cells that had adhered to the underside of the membrane were fixed, stained with Coomassie Brilliant Blue and counted under a dissecting microscope.

### *In vivo* experiments

NOD-SCIDγ_c_^null^ (NSG) mice were bred and maintained at the Toronto Medical Discovery Tower, MaRs Centre (Toronto, ON, Canada). Female mice (8–12 weeks old) were injected with 5 × 10^6^ breast cancer cells in 100 μl phosphate-buffered saline into the mammary fat pad. At day 21, local tumors were measured in two dimensions by calipers and the mice were killed. To enumerate lung metastases, lungs were fixed in 4% paraformaldehyde and tumor nodules were counted under a dissecting microscope as described before.^51^ Animal protocols were approved by the Sunnybrook Research Institute animal care committee.

### Statistical analysis

All *in vitro* experiments were performed in triplicate and repeated three times. Data are presented as mean±standard error unless otherwise indicated. Unpaired two-tailed student *t*-tests were used to determine *P*-values for differences between sample means. *P*-values less than 0.05 were considered significant.
